# Changes in treatment strategy over time for arteriovenous malformation in a Japanese high-volume center

**DOI:** 10.1186/s12883-020-01987-8

**Published:** 2020-11-05

**Authors:** Katsuya Komatsu, Yasushi Takagi, Akira Ishii, Takayuki Kikuchi, Yukihiro Yamao, Kazumichi Yoshida, Susumu Miyamoto

**Affiliations:** Department of Neurosurgery, Kyoto University Graduate School of Medicine, 54 Shogoin Kawahara-cho, Sakyo-ku, Kyoto, 606-8507 Japan

**Keywords:** Arteriovenous malformation, Post-ARUBA trial, Change in treatment strategy

## Abstract

**Background:**

Despite rapid developments in devices used to treat arteriovenous malformation (AVM), a randomised trial of Unruptured Brain Arteriovenous malformations published in 2014 recommended conservative treatment for nonhemorrhagic AVM. The purpose of the current retrospective study was to confirm how AVM treatment in Japan has changed and to assess the safety of treatment for hemorrhagic and nonhemorrhagic AVMs.

**Methods:**

We enrolled 242 consecutive patients with AVM; each patient’s treatment was selected and performed at our hospital. The type of onset, Spetzler–Martin (S–M) grade, age, sex, selected treatment, mortality, and morbidity were compared between the first and second periods of our study.

**Results:**

In patients with grade I–III AVM, the selected treatment changed between the first and second periods; however, in grade IV and V patients, the selected treatment did not change. Overall, interventions by microsurgery alone decreased (*p* < 0.001), the proportion of total treatments including microsurgery decreased (*p* = 0.005), interventions using stereotactic radiosurgery (SRS) alone increased (*p* = 0.009), and interventions including SRS increased (*p* = 0.002). Morbidity associated with intervention was 0.92% in the first period and 0% in the second period, and mortality was 0.92% in the first period and 1.67% in the second.

**Conclusions:**

With the development of new devices, the selected treatment was changed in patients with S–M grade I–III AVM, but was not changed in patients with grade IV and V. The complication rate was low and did not change throughout the periods. These findings suggest that the safety of treatment depends on a full understanding of device development and the selection of proper treatment, not on hemorrhagic onset. Further treatment innovations are expected to change the treatment for grade IV and V AVMs.

## Background

The results of A Randomised trial of Unruptured Brain Arteriovenous malformations (ARUBA) published in 2014 [1] offered the opportunity to reconsider the treatment strategy for nonhemorrhagic-onset brain arteriovenous malformation (AVM). In regard to treatment interventions for nonhemorrhagic cases, the ARUBA trial [1] and Scottish Audit [2] reported that intervention for nonhemorrhagic AVMs should be approached cautiously. Microsurgery, endovascular embolization, and stereotactic radiosurgery are combined therapies for AVM. Recently, studies have evaluated Onyx (Onyx® Liquid Embolic System; eV3 Inc., Irvine, CA) for use in endovascular embolization [3, 4], and multiple irradiation in gamma knife surgery for use in radiation therapy [5]. AVM factors such as Spetzler–Martin grade (S–M grade) [6], the type of onset, location and size of the nidus, and the presence of deep drainage veins affect the selection of the AVM treatment strategy. There are no recent Japanese data showing how high the risk of intervention for hemorrhagic and non-hemorrhagic AVM is. Therefore, this study aimed to evaluate the changes in the selection of AVM treatment strategy as devices have evolved, and to assess the treatment safety for hemorrhagic and nonhemorrhagic AVMs in Japan.

## Methods

Patients with AVM admitted to and hospitalized in our facility underwent thorough examinations, and each patient’s treatment strategy was determined during a multidisciplinary meeting based on the examination results. Between January 1998 and September 2017, we determined the treatment strategy for 242 consecutive patients with AVM. The number of patients per S–M grade I–V was 42, 79, 61, 45, and 15, respectively. Since May 2009, we have used the same decision-making process; therefore, we divided patients into two groups (first period: January 1998–April 2009 [154 patients]; second period: May 2009–September 2017 [88 patients]). In the first period, embolization was performed using NBCA or coils, and radiation therapy was mainly performed using X-knife or Novalis. In the second period, embolization was mainly performed using Onyx, and radiotherapy was performed using the Gamma knife. Intervention or no intervention, selected treatment, and type of onset (hemorrhagic vs. nonhemorrhagic) were compared between the first and second periods of our study. Morbidity was defined by the ARUBA trial criteria and neurological deterioration graded by a modified Rankin Scale (mRS) score ≥ 2. Patient backgrounds and characteristics are shown in Table 1.

**Table 1:** Patients Characteristics

	(*n* = 154)	(*n* = 88)	(*n* = 242)	*p* value
	First period	Second period	Total	
No. of Pt.				
Gr. I n (%)	29 (18.8)	13 (18.8)	42 (17.4)	0.483
Gr. II n (%)	46 (29.9)	33 (37.5)	79 (32.6)	0.255
Gr. III n (%)	39 (25.3)	22 (25.0)	61 (25.2)	1.000
Gr. IV n (%)	31 (20.1)	14 (15.9)	45 (18.6)	0.493
Gr. V n (%)	9 (5.8)	6 (6.8)	15 (6.2)	0.786
Age	33.1 ± 16.3	33.3 ± 17.5		0.219
Sex(M, F)	82, 72	46, 42	128, 114	0.894
Onset n (%)				
Hemorrhage	78 (50.6)	36 (40.9)	114 (47.1)	0.181
Seizure	30 (19.5)	17 (19.3)	47 (19.4)	1.000
Incidental	21 (13.6)	14 (15.9)	35 (14.5)	0.705
Headache	18 (11.7)	18 (20.5)	36 (14.9)	0.090
Cranial nerve palsy	4 (2.6)	2 (2.3)	6 (2.5)	1.000
other	3 (1.9)	1 (1.1)	4 (1.7)	1.000
Interventional cases (%)				
Gr. I	24 (82.8)	13 (100)	37 (88.1)	0.302
Gr. II	43 (93.5)	28 (84.8)	71 (89.9)	0.268
Gr. III	30 (76.9)	14 (63.6)	44 (72.1)	0.373
Gr. IV	10 (32.3)	4 (28.6)	14 (31.1)	1.000
Gr.V	2 (22.2)	1 (16.7)	3 (20.0)	1.000
	109	60	169	0.666
Treatment n (%)				
surgery (S) only	73 (47.4)	11 (12.5)	84 (34.7)	< 0.001
S + embolization (E)	18 (11.7)	23 (26.1)	41 (16.9)	0.007
S + radiation (R)	6 (3.9)	4 (4.5)	10 (4.1)	1.000
S + E + R	1 (0.6)	1 (1.1)	2 (0.8)	1.000
R only	8 (5.2)	14 (15.9)	22 (9.1)	0.009
E + R	1 (0.6)	4 (4.5)	5 (2.1)	0.060
E only	2 (1.3)	3 (3.4)	5 (2.1)	0.357
medical	45 (29.2)	28 (31.8)	73 (30.2)	0.673
include surgery	98 (63.6)	39 (44.3)	137 (56.6)	0.005
include radiation	16 (10.4)	23 (26.1)	39 (16.1)	0.002
mortbidity, n (%)	1 (0.92)	0 (0)	1 (0.59)	1.000
mortality, n (%)	1 (0.92)	1 (1.67)	2 (1.18)	1.000

Baseline characteristics of patients with AVMs and the proportion of each treatment for each S–M grade during the first and second periods of our study

### The current fundamental treatment policy for AVM

The current fundamental treatment policy for AVM at our facility is determined by the following three elements: (1) Whether the lesion is in the eloquent area, (2) whether it is symptomatic prior to intervention, and (3) whether the intervention could disturb consciousness. In addition, if the lesion is capable of embolization, we actively chose treatment including embolization. For patients with AVM located in the eloquent area, hemorrhagic onset, and symptoms, treatment including microsurgery was performed. If there were no symptoms, AVM located in the eloquent area, and hemorrhagic onset, treatment without microsurgery was considered. For patients with AVM located in the eloquent area, treatment without microsurgery was considered if the intervention could disturb consciousness. For patients with AVM located in the non-eloquent area and hemorrhagic onset, treatment including microsurgery was performed. For patients with AVM located in the eloquent area and nonhemorrhagic onset, embolization and stereotactic radiosurgery were considered. For non-eloquent AVM and nonhemorrhagic onset, combined treatment including microsurgery was performed. For nonhemorrhagic onset with AVM located in the visual cortex, intervention was undertaken if the risk of visual field impairment was acceptable to the patient. Regardless of the presence of hemorrhagic onset or symptoms, treatment for high S–M grade AVM should be fully considered.

### Ethical approval

This study adhered to the principles set forth in the US Code of Federal Regulations, Title 45, Part 46, Protection of Human Subjects, revised January 15, 2009 (http://www.hhs.gov/ohrp/humansubjects/guidance/45cfr46.html) and the World Medical Association Declaration of Helsinki (http://www.wma.net/en/30publications/10policies/b3/index.html). This study was approved by the institutional review board of Kyoto University (R2088–2) with a waiver of individual consent.

### Statistical analysis

Data are expressed as the mean ± standard deviation. Fisher’s exact probability test was used to compare the selected treatment, onset, morbidity, and mortality between the first and second periods, and between hemorrhagic and nonhemorrhagic groups. All statistical analyses were performed using SPSS software (version 22; IBM, Armonk, NY, USA). A *p*-value less than 0.05 was considered to indicate statistical significance.

## Results

Comparing the first period with the second period, the number of patients with each S–M grade was as follows: grade I: 29 vs. 13 patients (total: 42 patients); grade II: 46 vs. 33 (total: 79); grade III: 39 vs. 22 (total: 61); grade IV: 31 vs. 14 (total: 45); and grade V: 9 vs. 6 (total: 15), respectively. The mean age was 33.1 ± 16.3 years in the first period and 33.3 ± 17.5 years in the second period. There were 82 men and 72 women in the first period, and 46 men and 42 women in the second period. The number of hemorrhagic-onset patients was 78 in the first period and 36 in the second period. There was no significant difference between periods for the proportion of patients with each S–M grade, age, sex, and hemorrhagic-onset.

The number of patients receiving intervention was 37 (88.1%) grade I, 71 (89.9%) grade II, 44 (72.1%) grade III, 14 (31.1%) grade IV, and 3 (20.0%) grade V. The proportion of hemorrhagic-onset patients was 50.0% (21/42) grade I; 51.9% (41/79) grade II; 45.9% (28/61) grade III; 42.2% (19/45) grade IV, and 33.3% (5/15) grade V, with 109 patients (70.8%) patients undergoing intervention in the first period and 60 patients (68.2%) undergoing intervention in the second period. There was no statistical difference in the intervention rate between the first and second periods, or for each grade.

Intervention by microsurgery alone decreased significantly comparing the first and second periods. The use of combined microsurgery and endovascular embolization increased significantly, and the use of stereotactic radiosurgery alone also increased significantly. Overall, interventions including microsurgery decreased significantly, and interventions including stereotactic radiosurgery increased significantly. The morbidity rate associated with intervention was 0.92% in the first period and 0% in the second period, and the mortality rate was 0.92% in the first period and 1.67% in the second period (Table 1).

### Comparison between patients with hemorrhagic and nonhemorrhagic AVM

We found no significant differences for S–M grade, and no difference in the proportion of each S–M grade, age, and sex when comparing hemorrhagic (*n* = 114) and nonhemorrhagic (*n* = 128) patients. Regarding the intervention rate, there was no difference for patients with S–M grade I; however, patients with S–M grade II had high intervention rates for both hemorrhagic (97.6%) and nonhemorrhagic (81.6%) AVM, but the rate of intervention was significantly lower in nonhemorrhagic AVM (*p* = 0.025). In S–M grade III and IV patients, the rate of intervention was significantly lower in nonhemorrhagic AVM (III: *p* = 0.044 and IV: *p* = 0.011, respectively). For S–M grade V patients, intervention was often difficult, and therefore there was no difference between hemorrhagic and nonhemorrhagic patients. Overall, there were significantly fewer interventions by microsurgery alone and fewer interventions including microsurgery for nonhemorrhagic AVM. The proportion of patients undergoing medical treatment only was significantly greater for nonhemorrhagic AVM. Morbidity associated with intervention was 1.05% (1/95 intervention cases) in hemorrhagic AVM and 0% (0/74) in nonhemorrhagic AVM. Mortality associated with intervention was 1.75% (2/95) in hemorrhagic AVM and 0% (0/74) in nonhemorrhagic AVM. There were no statistical differences in morbidity and mortality between patients with hemorrhagic and nonhemorrhagic AVM (Table 2).

**Table 2:** Hemorrhagic vs nonhemorrhagic AVM

	(*n* = 114)	(*n* = 128)	*p* value
	Hemorrhagic	Non-hemorrhagic	
No. of Pt.			
Gr. I n (%)	21 (18.4)	21 (16.4)	0.735
Gr. II n (%)	41 (36.0)	38 (29.7)	0.337
Gr. III n (%)	28 (24.6)	33 (25.8)	0.883
Gr. IV n (%)	19 (16.7)	26 (20.3)	0.511
Gr. V n (%)	5 (4.4)	10 (7.8)	0.299
Age	31.7 ± 16.7	34.4 ± 16.6	0.219
Sex(M, F)	57, 57	70, 58	0.520
Interventional cases (%)			
Gr. I	19 (90.5)	18 (85.7)	1.000
Gr. II	40 (97.6)	31 (81.6)	0.025
Gr. III	24 (85.7)	20 (60.6)	0.044
Gr. IV	10 (52.6)	4 (15.4)	0.011
Gr.V	2 (40.0)	1 (10.0)	0.242
	95	74	
Treatment n (%)			
surgery (S) only	54 (47.4)	30 (23.4)	< 0.001
S + embolization (E)	15 (13.2)	26 (20.3)	0.170
S + radiation (R)	9 (7.9)	1 (0.8)	0.007
S + E + R	1 (0.9)	1 (0.8)	1.000
R only	10 (8.8)	12 (9.4)	1.000
E + R	3 (2.6)	2 (1.6)	0.669
E only	3 (2.6)	2 (1.6)	0.669
medical	19 (16.7)	54 (42.2)	< 0.001
include surgery	79 (69.3)	58 (45.3)	< 0.001
include radiation	23 (20.2)	16 (12.5)	0.117
mortbidity, n (%)	1 (1.05)	0 (0)	1.000
mortality, n (%)	2 (1.75)	0 (0)	0.505

Baseline characteristics of patients with hemorrhagic vs nonhemorrhagic AVM

### Morbidity and mortality

Only one patient in the first period developed neurological deterioration with an mRS score ≥ 2 following intervention. Two patients died after intervention, both of which had S–M grade IV AVM with repeated hemorrhage; one patient died in the first period, and the other patient died in the second period. Although the deterioration was not mRS ≥ 2, six patients developed visual field defects. Overall morbidity associated with intervention was 0.92% (1/109 intervention cases) in the first period and 0% (0/60 intervention cases) in the second period. Mortality associated with intervention was 0.92% (1/109) in the first period and 1.67% (1/60) in the second period.

### Comparing treatment for patients with each S–M grade between the first and second periods

#### Grade I

**Fig. 1:**
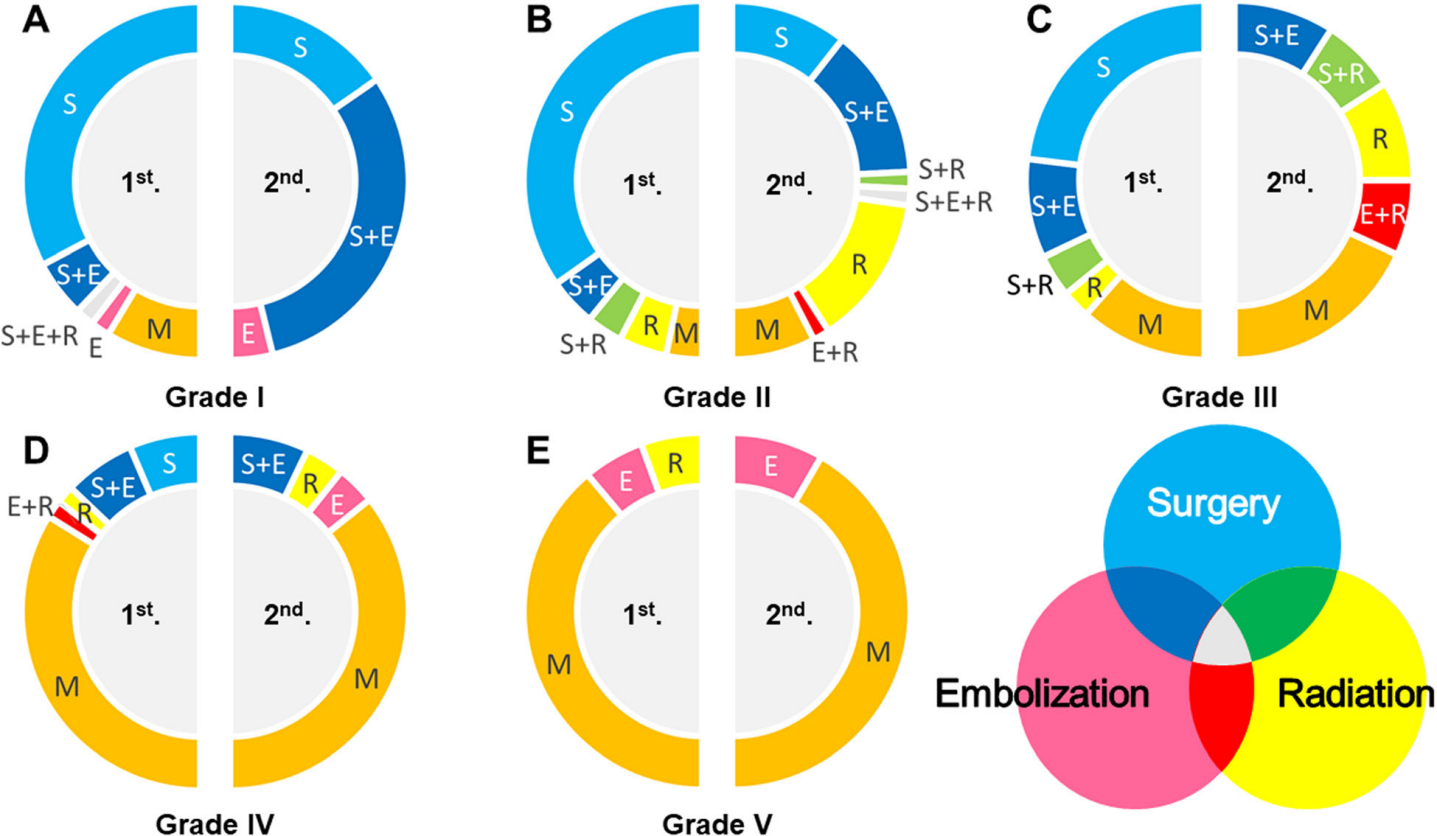
The proportions of each treatment for each S–M grade of AVM in the first and second periods of our study: grade I (**a**), grade II (**b**), grade III (**c**), grade IV (**d**), and grade V (**e**). S: microsurgery, E: endovascular embolization, R: stereotactic radiosurgery, M: medical treatment

Patients with S–M grade I AVMs were an almost homogeneous group. We intervened in 24/29 patients (82.8%) in the first period and all 13 patients (100%) in the second period. Among patients undergoing intervention, treatment included microsurgery in 23 patients in the first period and 12 patients in the second period. Treatment with microsurgery alone was conducted in 19 patients in the first period and 4 patients in the second period. The use of microsurgery alone decreased significantly as a treatment strategy (*p* = 0.049), and the combination of microsurgery and endovascular embolization increased significantly (*p* < 0.001). Although a high proportion of patients with S–M grade I AVMs underwent treatments including microsurgery, the number of microsurgical procedures increased after the introduction of endovascular embolization (Fig. 1a). Mortality and morbidity rates were both 0% following intervention for grade I AVMs throughout the study.

#### Grade II

**Fig. 2:**
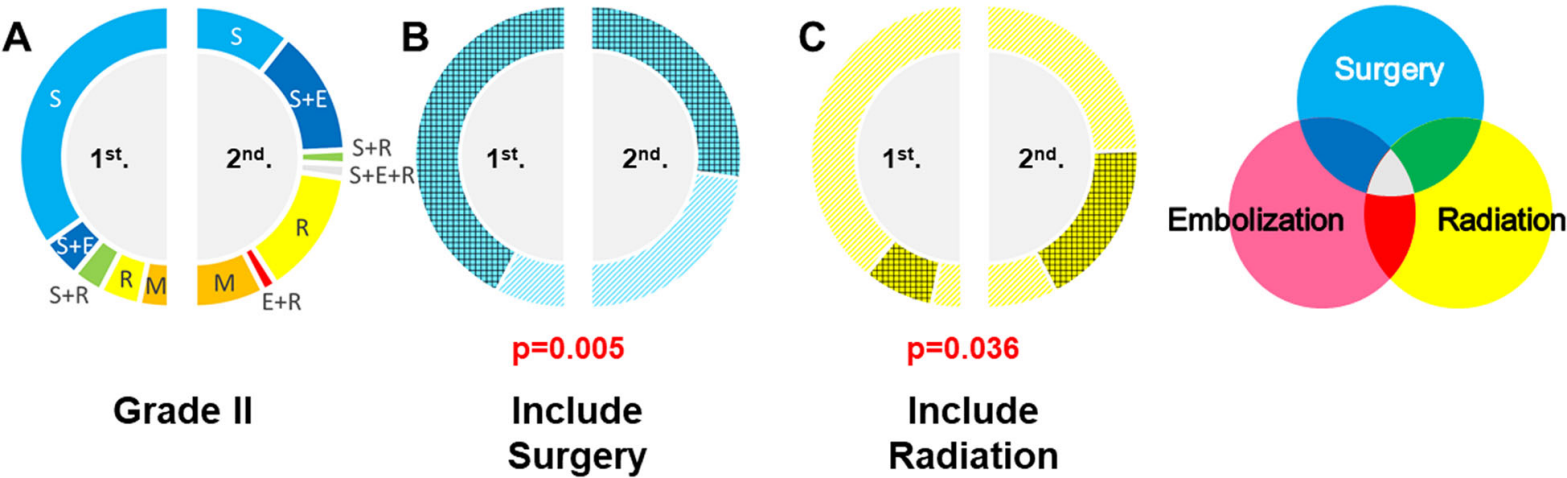
The proportion of each treatment for patients with S–M grade II AVMs in the first and second periods of our study (**a**). The blue gridded area indicates the proportion of treatments including microsurgery (**b**), and the yellow gridded area indicates the proportion of treatments including stereotactic radiosurgery (**c**). Treatments including microsurgery decreased (*p* = 0.005) and treatments including stereotactic radiosurgery increased (*p* = 0.036) when comparing the first and second periods in our study. S: microsurgery, E: endovascular embolization, R: stereotactic radiosurgery, M: medical treatment

In patients with S–M grade II AVMs, the treatment strategy changed from the first to the second periods (Fig. 1b). The rate of microsurgery alone decreased (*p* < 0.001) (Fig. 2a), the combination of microsurgery and endovascular embolization increased (*p* = 0.035) (Fig. 2a), stereotactic radiosurgery alone increased (*p* = 0.035) (Fig. 2a), treatment including microsurgery decreased (*p* = 0.005) (Fig. 2b), and treatment including stereotactic radiosurgery increased (*p* = 0.036) (Fig. 2c).

Patients with S–M grade II AVMs appeared as three subtypes. The first group had a medium-sized nidus (3–6 cm) (size 2, eloquent 0, deep drainage 0: S2E0D0); the second group had a small-sized (< 3 cm) nidus located in the eloquent area (S1E1D0); and the third group had deep venous drainage with a small-sized nidus (S1E0D1).

**Fig. 3:**
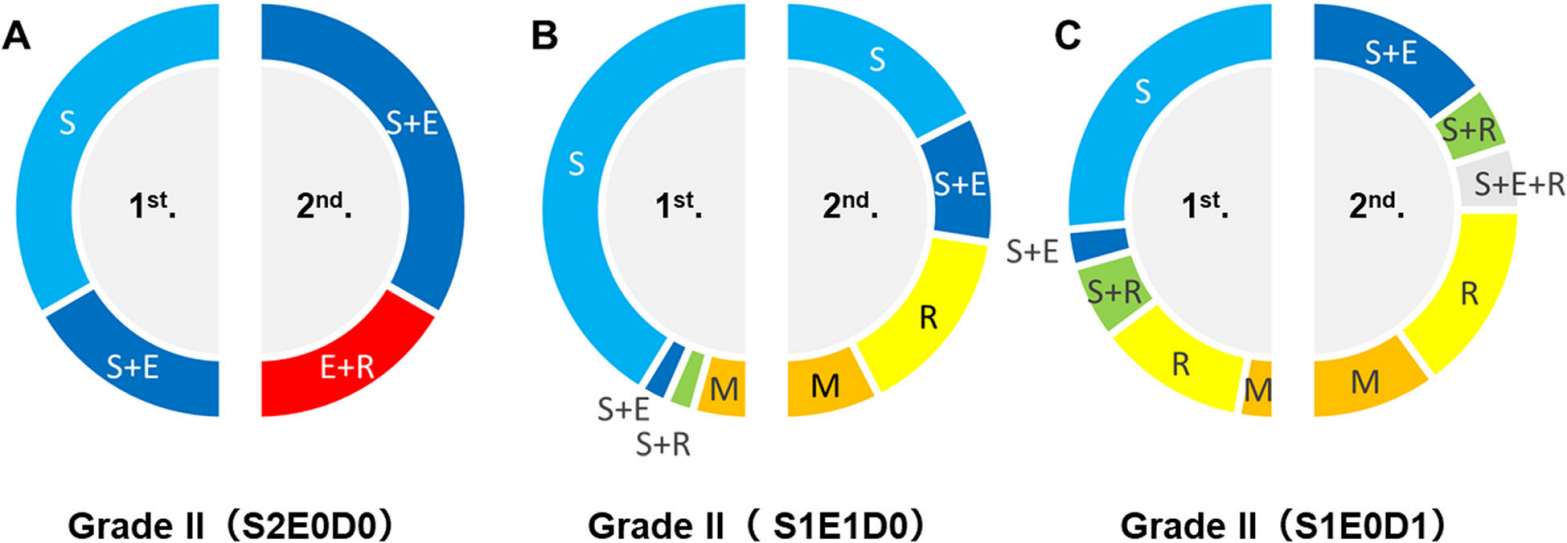
The proportion of each treatment for each subtype of S–M grade II AVM in the first and second periods of our study: S2E0D0 subtype (**a**), S1E1D0 subtype (**b**), and S1E0D1 subtype (**c**)

In the S2E0D0 subgroup, there was no significant change in the selected treatment (Fig. 3a). In the S1E1D0 subgroup, the rate of microsurgery alone decreased significantly (*p* = 0.002) (Fig. 3b); however, the rates of stereotactic radiosurgery alone and treatment including stereotactic radiosurgery increased significantly (*p* = 0.006, 0.038) (Fig. 3b). In the S1E0D1 subgroup, treatment with microsurgery alone decreased significantly (*p* = 0.009) (Fig. 3c). Only one patient with hemorrhagic-onset grade II AVM experienced neurological deterioration after microsurgery during the first period, thus mortality and morbidity associated with intervention were 0 and 2.3% (1/43 intervention cases) in the first period, respectively. In the second period, mortality and morbidity according to the intervention were both 0%.

#### Grade III

**Fig. 4:**
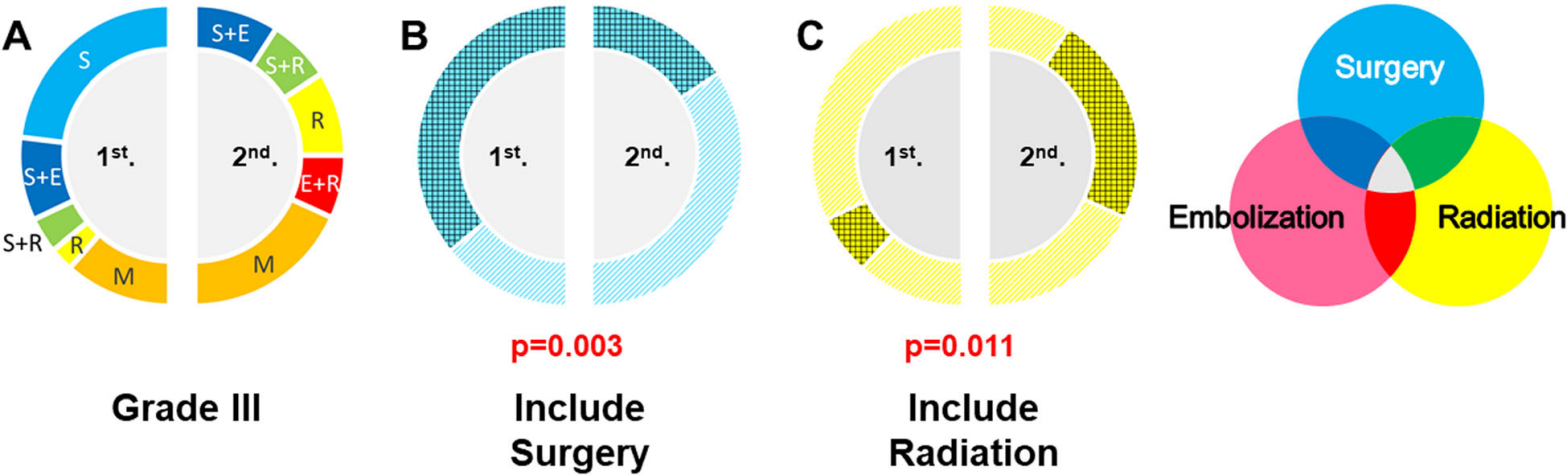
The proportion of each treatment for patients with S–M grade III AVMs in the first and second periods of our study (**a**). The blue gridded area indicates the proportion of treatments including microsurgery (**b**), and the yellow gridded area indicates the proportion of treatments including stereotactic radiosurgery (**c**). Treatments including microsurgery decreased significantly (*p* = 0.003), and treatments including stereotactic radiosurgery increased significantly (*p* = 0.011) when comparing the first and second periods of our study. S: microsurgery, E: endovascular embolization, R: stereotactic radiosurgery, M: medical treatment

In patients with S–M grade III AVMs, microsurgery alone decreased significantly (*p* < 0.001) (Fig. 1c and 4a), the combination of endovascular embolization and stereotactic radiosurgery increased significantly (*p* = 0.043) (Fig. 4a), treatment including microsurgery decreased significantly (*p* = 0.003) (Fig. 4b), and treatment including stereotactic radiosurgery increased significantly (*p* = 0.011) (Fig. 4c).

**Fig. 5:**
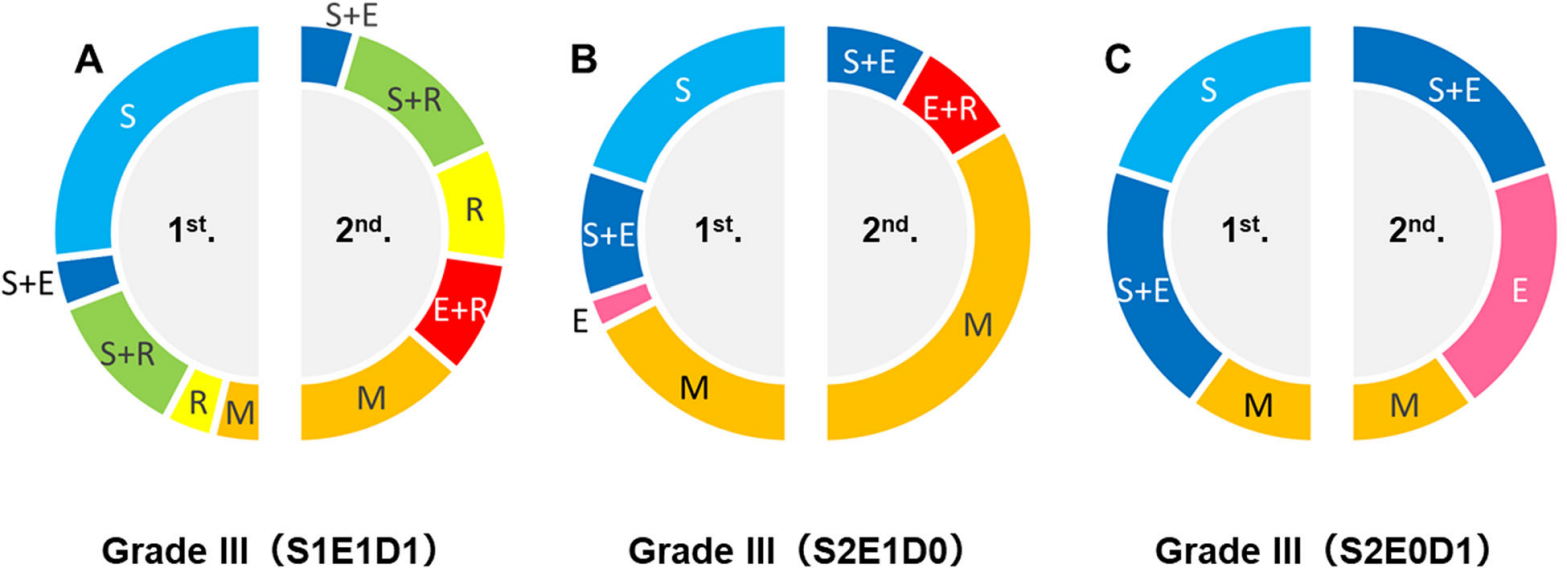
The proportion of each treatment for each subtype of S–M grade III AVMs in the first and second periods of our study: S1E1D1 subtype (**a**), S2E1D0 subtype (**b**), and S2E0D1 subtype (**c**)

Patients with grade III AVMs were subdivided into four subtypes. The first group had a small-sized nidus in the eloquent area with deep venous drainage (S1E1D1); the second group had a medium-sized nidus located in the eloquent area (S2E1D0); the third group had deep venous drainage with a medium-sized nidus (S2E0D1); and the fourth group had a large-sized nidus (S3E0D0). We saw only one patient with the S3E0D0 subtype in the first period and none in the second period; therefore, comparisons between periods were not possible. In the S1E1D1 subtype, microsurgery alone decreased significantly (*p* = 0.006) (Fig. 5a), and treatment including microsurgery also decreased significantly (*p* = 0.033) (Fig. 5a). For the other subgroups, the number of patients was small, and we saw no significant difference as a result (Fig. 5b and c). Both mortality and morbidity according to intervention were 0% throughout the study period.

#### Grade IV

In patients with S–M grade IV AVMs, we saw no change in the selected treatment between the two periods. Patients with S–M grade IV AVMs were divided into three subtypes. The first group had a medium-sized nidus in the eloquent area with deep venous drainage (S2E1D1); the second group had a large-sized nidus located in the eloquent area (S3E1D0); and the third group had a large-sized nidus with deep venous drainage (S3E0D1). The S2E1D1 subgroup consisted of a relatively high number of patients; however, the treatment strategy remained unchanged. The other subgroups consisted of only a small number of patients, and no statistical difference was observed (Fig. 1d). Although no patients developed neurological deterioration in either period, two patients developed repeated hemorrhagic leading to death despite intervention (1 patient in the first period and 1 patient in the second period). Mortality and morbidity according to intervention were, respectively, 10.0% (1/10) and 0% in the first period, and 25.0% (1/4) and 0% in the second period, respectively.

#### Grade V

In patients with S–M grade V AVMs, we saw no significant difference in the selected treatment between the first and second periods. Microsurgery was not selected in either period, and medical treatment was selected for 80% of patients. Among 15 patients with S–M grade V AVMs, only three patients underwent intervention (2 underwent endovascular embolization alone and 1 underwent stereotactic radiosurgery alone; Fig. 1e). No patients developed neurological deterioration secondary to intervention, and no patients died. Both mortality and morbidity according to intervention were 0% throughout the study period.

## Discussion

Comparing between the first and second periods, overall cases showed that the intervention by microsurgery alone decreased, the proportion of total treatments including microsurgery decreased, the intervention by stereotactic radiosurgery alone increased, and the intervention including stereotactic radiosurgery also increased. Complications associated with the intervention were acceptable in both the first and second periods. With the development of gamma knife treatment [7, 8] and endovascular embolization represented by Onyx [3, 4], this result demonstrates that safe therapy is possible by combining various modalities when developing strategy for AVM.

In patients with S–M grade II and III AVM, the selected treatment was particularly affected by developments in stereotactic radiotherapy and endovascular embolization. Although there were different changes in treatment combinations for each subtype in the same grade, S-M grade II and III AVM was treated safely. This result shows that safer AVM treatment can be achieved by combining the advantages of each treatment throughout the entire period.

The S–M grade is an excellent scale that expresses the surgical risk easily and practically based on the three elements [6]. Grade III was the most heterogeneous group, and because of the different elements within the same grade, it became a group with uneven surgical risk. In modified S–M grade, which classifies grade III subtypes in detail, the surgical risk is evaluated for each subtype [9]. Small-sized nidus (S1E1D1) reported the lowest surgical risk, and medium-sized nidus located in the eloquent area (S2E1D0) reported the highest surgical risk [9]. Large grade III (S3E0D0) was reported to be extremely rare [9]. In addition, supplemental S–M grade was proposed to accurately predict surgical risk [10]. The small-sized grade III AVM (S1E1D1) was classified as low risk, and the medium-sized one located in the eloquent area (S2E1D0) was classified as high risk [10]. These reports demonstrate that S1E1D1 and S2E1D0 have different surgical risks in the same S–M grade III. In our study, there were no cases that were exacerbated by intervention regardless of the presence or absence of hemorrhagic onset in grade III AVM. There were 24 cases of small-sized nidus classified as low risk by supplemental S–M grade (20 received intervention; 83.3%), and 37 cases of medium-sized or larger nidus classified as high risk (24 received intervention; 64.9%). This result revealed that safe interventions were performed even in cases with medium- or larger-sized nidus without aggravating those who chose intervention.

Furthermore, there was no change in the selected treatment for grade IV and V AVM between the first and second periods. Therefore, it was considered that the development of endovascular embolization and stereotactic radiosurgery mainly benefited grade II and III AVM, and did not contribute to grade IV and V. Even with device evolution, there was not enough power to change the treatment strategy for grade IV and V AVM.

Comparing complications in our study with those reported in the ARUBA trial, there was no difference in complication rate for intervention in nonhemorrhagic S–M grade I AVM (*p* = 0.144, data not shown); however, there were significantly fewer complications in our patients with grade II and III AVM (*p* < 0.001, *p* < 0.001, respectively, data not shown). Interventions for low-grade AVMs were associated with fewer complications in previous studies and in our study. All patients experiencing complications associated with the intervention had hemorrhagic onset, and none experienced complications associated with the intervention for nonhemorrhagic onset in our study [7, 11–15]. We also found that intervention for S–M grade I–III AVM provided good results regardless of the type of AVM onset.

Furthermore, when comparing the selected treatment of our study with the Scottish Audit [2], the proportion of each S–M grade was equivalent to that in our institution; however, the selected treatment was significantly different from ours. In the Scottish Audit, there were significantly fewer selected treatments including microsurgery (*p* < 0.001), and embolization alone and the combination of embolization and stereotactic radiosurgery were significantly greater compared with ours (*p* < 0.001, *p* = 0.001, data not shown). The AHA/ASA scientific statement noted the problems of the ARUBA trial and the Scottish Audit [16]. Clinicians are urged to consider both the risk of future bleeding associated with unruptured and ruptured AVM and the risk of the treatment itself. These statements emphasized that appropriate intervention should be undertaken for AVMs for which intervention was considered safe, and that medical treatment should be selected for patients with nonhemorrhagic-onset AVMs who were at risk for complications as a result of the intervention. These findings suggest that the most important factor in the treatment for AVM is the proper selection of treatments for safer intervention, not hemorrhagic onset. The changes in selected treatments between the first and second periods are likely the result of a better understanding of device development, aiming for safer treatment.

### Limitations

All patients with AVM presenting to our hospital were hospitalized for examination, and the treatment strategy was determined in a multidisciplinary meeting. Outcome data in patients not undergoing intervention were not available because these patients were not followed at our hospital; we only had data for patients undergoing intervention. Therefore, we do not know the subsequent event rate in patients with AVM undergoing medical treatment only.

Although the overall number of patients in our study was reasonable, studies with higher numbers of patients are needed because the number of patients was low when comparing the two periods and each subtype.

## Conclusion

We compared selected treatments for AVM in two periods. In patients with S–M grade I–III AVM, the selected treatment was affected by device development. The selected treatment for patients with S–M grade IV and V AVM remained the same. The complication rate did not change throughout the periods. The results suggest that the safety of treatment depends on a better understanding of device development and proper treatment selection, not on hemorrhagic onset. Further treatment innovations are expected to change the treatment for grade IV and V AVMs.

## Data Availability

The datasets analyzed during the current study are available from the corresponding author upon request.

## Abbreviations

AVM: Arteriovenous malformation
S-M: Spetzler–Martin
ARUBA: A Randomised trial of Unruptured Brain Arteriovenous malformations
mRS: Modified Rankin Scale
